# Cross-Reactivity of Neutralizing Antibodies among Malignant Catarrhal Fever Viruses

**DOI:** 10.1371/journal.pone.0145073

**Published:** 2015-12-14

**Authors:** Naomi S. Taus, Cristina W. Cunha, Jana Marquard, Donal O’Toole, Hong Li

**Affiliations:** 1 Animal Disease Research Unit, Agricultural Research Service, United States Department of Agriculture, Pullman, Washington, United States of America; 2 Department of Veterinary Microbiology and Pathology, Washington State University, Pullman, Washington, United States of America; 3 Wyoming State Veterinary Laboratory, University of Wyoming, Laramie, Wyoming, United States of America; The University of Melbourne, AUSTRALIA

## Abstract

Some members of the gamma herpesvirus genus *Macavirus* are maintained in nature as subclinical infections in well-adapted ungulate hosts. Transmission of these viruses to poorly adapted hosts, such as American bison and cattle, can result in the frequently fatal disease malignant catarrhal fever (MCF). Based on phylogenetic analysis, the MCF viruses (MCFV) cluster into two subgroups corresponding to the reservoir hosts’ subfamilies: Alcelaphinae/Hippotraginae and Caprinae. Antibody cross-reactivity among MCFVs has been demonstrated using techniques such as enzyme linked immunosorbent and immunofluorescence assays. However, minimal information is available as to whether virus neutralizing antibodies generated against one MCFV cross react with other members of the genus. This study tested the neutralizing activity of serum and plasma from select MCFV-infected reservoir hosts against alcelaphine herpesvirus 1 (AlHV-1) and ovine herpesvirus 2 (OvHV-2). Neutralizing antibody activity against AlHV-1 was detected in samples from infected hosts in the Alcelaphinae and Hippotraginae subfamilies, but not from hosts in the Caprinae subfamily. OvHV-2 neutralizing activity was demonstrated in samples from goats (Caprinae) but not from wildebeest (Alcelaphinae). These results show that neutralizing antibody cross reactivity is present to MCFVs within a virus subgroup but not between subgroups. This information is important for diagnosing infection with MCFVs and in the development of vaccines against MCF.

## Introduction

The gamma herpesvirus genus *Macavirus* currently contains 10 viruses also referred to as malignant catarrhal fever viruses (MCFV) as well as lymphotropic herpesviruses of various species [1, 2]. The MCFVs are maintained as life-long sub-clinical infections in well-adapted reservoir hosts in the sub-families Alcelaphinae, ex. wildebeest (*Connochaetes sp*.), Hippotraginae, ex. roan antelope (*Hippotragus equinus*), and Caprinae, ex. sheep (*Ovis aries*) and goats (*Capra hircus*). Transmission of some of these viruses to poorly-adapted hosts such as domestic cattle and bison (Bovidae) and deer (Cervidae) results in the frequently fatal disease syndrome known as malignant catarrhal fever (MCF). Losses from MCF for producers of highly susceptible species such as bison can be substantial [3, 4]. Currently there is no vaccine to protect against MCF and treatment, when attempted, is limited to supportive care, which is largely unsuccessful.

Based on phylogenetic analysis of a portion of the viral DNA polymerase gene the MCFVs can be divided into two subgroups corresponding to their reservoir hosts: Alcelaphinae/Hippotraginae and Caprinae [5]. Alcelaphine herpes virus-1 (AlHV-1), which is carried by wildebeest (Alcelaphinae), and ovine herpesvirus 2 (OvHV-2), carried by sheep (Caprinae) are the most extensively studied MCFVs. Comparison of AlHV-1 and OvHV-2 genomes showed 62 open reading frames (ORFs) conserved with other gammaherpesvirus, ten ORFs present only in these two viruses, two ORFs unique to AlHV-1, and three ORFs unique to OvHV-2 [6, 7].

MCFVs in both Alcelaphinae/Hippotraginae and Caprinae subgroups contain a conserved epitope that can be detected serologically using a monoclonal antibody, 15-A, in a competitive inhibition ELISA (cELISA) [8]. The epitope recognized by 15-A is unknown but is present in a glycoprotein complex of 115, 110, 105, 78, and 45 kDa which is immunoprecipitated from AlHV-1-infected cell lysates [9]. The antibody recognizes a 45kDa protein in immunoblots of purified AlHV-1 virions [9]. Although neutralizing antibodies against AlHV-1 have been detected in MCFV-infected wildebeest and hartebeest, as well as other species in the Alcelaphinae and Hippotraginae subfamilies [10, 11], the extent of neutralizing antibody cross-reactivity between the MCFVs, particularly those carried by species in the Caprinae subfamily, has not been extensively studied. This is partly because most of the MCFVs have not been cultured *in vitro*. AlHV-1 can be cultured *in vitro* so it is possible to assess neutralizing antibody cross-reactivity to AlHV-1 from animals infected with other MCFVs. However, OvHV-2 cannot be cultured *in vitro* so standard antibody neutralization testing cannot be used. Recently, an *in vivo* system, using rabbits as a model, has been developed to test virus neutralizing antibody reactivity against OvHV-2 [12]; although this system is not practical for diagnostic purposes, it is valuable for testing cross-reactivity of MCFV antibodies against OvHV-2.

The aim of this study was to determine whether infection with various MCFVs resulted in antibodies that had cross-reactive neutralizing activity to AlHV-1 and OvHV-2. Knowledge about neutralizing antibody cross-reactivity to MCFVs will help determine whether multiple vaccines need to be developed to protect against MCF caused by the various members of the MCFV group and clarify under what circumstances the AlHV-1 neutralization assay can be useful.

## Materials and Methods

### Serum and plasma for neutralization assays

Samples of serum or plasma, previously determined to be positive or negative for the presence of MCFV-specific antibodies, from an archive of various animal species (Table 1) stored at the Animal Diseases Research Unit -Agricultural Research Service- United States Department of Agriculture in Pullman, WA, were combined and re-assayed for titration of MCFV antibodies using cELISA as described [13]. This assay uses a monoclonal antibody, 15-A, which recognizes a conserved epitope present in all MCFVs examined to date. The highest dilution of each sample pool that showed ≥25% inhibition, the cut-off point for the assay, was determined (Table 1). Any sample pool showing < 25% inhibition at a 1:5 dilution was considered negative.

**Table 1:** Pooled serum and plasma samples used for virus neutralization assays.

MCFV reservoir host subfamily	Species of each pool tested for neutralizing antibody (number of samples in pool)	MCFV infection status	Endpoint titers of plasma and serum pools		OvHV-2 neutralization^a^
			cELISA^b^	AlHV-1 neutralization^c^	
Caprinae	Domestic sheep^d^ (4)	Infected	320	8	6/6
	Domestic sheep (5)		320	32	
	Domestic sheep-Dorset (4)		160	32	
	Domestic goat^d^ (5)		320	32	4/6
	Domestic goat (4)		160	8	
	Barbary sheep (4)		40	32	
	Nubian Ibex (5)		160	16	
	Nubian Ibex (3)		80	8	
	Mouflon sheep (4)		160	16	
	Bighorn sheep (3)		8	16	
	Domestic sheep^d^ (5)	Uninfected	<5	4	0/6
	Domestic sheep (5)		<5	8	
	Domestic sheep-Dorset (4)		<5	16	
	Domestic goat^d^ (4)		<5	16	0/6
	Domestic goat (5)		<5	8	
	Barbary sheep (4)		<5	32	
	Nubian Ibex (4)		<5	8	
	Nubian Ibex (5)		<5	8	
	Mouflon sheep (5)		<5	16	
	Bighorn sheep (3)		<5	32	
Alcelaphinae	Wildebeest^d^ (4)	Infected	320	512	0/6
	Wildebeest (5)		320	512	
	Hartebeest (4)		40	256	
	Wildebeest^d^ (4)	Uninfected	<5	16	0/6
	Wildebeest (4)		<5	16	
Hippotraginae	Gemsbok (5)	Infected	320	512	
	Gemsbok (4)	Uninfected	<5	32	

^a^Number of surviving rabbits/Number of total rabbits.

^b^Titer is the reciprocal of the highest dilution which gave ≥ 25% inhibition, the minimum cut-off for the MCFV competitive inhibition ELISA.

^c^Titer is the reciprocal of the highest dilution which inhibited cytopathic effect in more than 50% of the wells at that dilution.

^d^Indicates pools used in both AlHV-1 and OvHV-2 neutralization assays.

### AlHV-1 neutralization assay

Fetal mouflon sheep kidney cells were grown in DMEM supplemented with 10% FBS, 100 units/ml of penicillin, 100μg/ml streptomycin, and 1μg/ml amphotericin B. Cells (2.5 x 10^4^ cells/well) and AlHV-1 (10^2^ TCID_50_) that had been incubated with two-fold serially diluted (1:8 to 1:512) sera or plasma for one hr at 37°C were mixed and then seeded in quadruplicate in 96-well microtiter plates. The plates were incubated at 37°C, 5% CO_2_ for five days. The titer of MCFV neutralizing Abs against AlHV-1 was the reciprocal of the highest serum or plasma dilution which inhibited cytopathic effect in more than 50% of the wells at that dilution.

### OvHV-2 neutralization assay

Twelve-week old New Zealand White (NZW) rabbits were used in the study. The rabbits were housed and handled in accordance with a protocol (#2232) approved by the Washington State University Institutional Animal Care and Use Committee. The rabbits were purchased from the Western Oregon Rabbit Company and compatible pairs were housed in 56”L x 56”W x 36”H cages in the vivarium at the College of Veterinary Medicine, Washington State University, Pullman, WA. Commercial laboratory rabbit feed was provided daily and hay was provided in plastic bottles for environmental enrichment. Water was available at all times via gravity feed bottles. Rabbits were euthanized by inducing a surgical plane of anesthesia using intramuscular injection of xylazine (5mg/kg) and ketamine (35mg/kg) followed by intracardiac injection of commercial barbiturate-based euthanasia solution (1ml/4.5kg).

#### OvHV-2 neutralizing activity of anti-AlHV-1 antibodies

OvHV-2 (1 ml) obtained from sheep nasal secretions as previously described [14] was incubated for one hr at 37°C with one ml of 1) pooled plasma from OvHV-2 uninfected sheep, negative for MCFV Ab (Ab-); 2) pooled plasma from OvHV-2 infected sheep, positive for MCFV Ab (Ab +); 3) pooled serum from AlHV-1 infected wildebeest, MCFV Ab+; or 4) pooled serum from uninfected wildebeest, MCFV Ab-, from the San Diego Zoological Wildlife Park. The MCFV Ab+ serum from infected wildebeest had a cELISA titer of 640. The sheep plasma cELISA endpoint titer of 320 was used to normalize the amount of wildebeest serum used for the experiment. Thus the final dilution of sheep plasma was 1:2 and wildebeest serum was 1:4. Rabbits (n = 24) were divided into four groups of six rabbits and inoculated by intranasal nebulization, as described [15], with two ml of antibody treated virus containing 10^6^ genome copies of OvHV-2. Following inoculation, blood was collected once or twice a week from each rabbit; DNA purified from peripheral blood leucocytes and plasma were stored at -20°C until used. Hemi-nested PCR was used to detect OvHV-2 DNA [15] and cELISA was used to monitor the development of MCFV antibody. Rabbits were observed daily for clinical signs and rectal temperatures were recorded daily beginning on day 16 post inoculation (PI). Rabbits were euthanized within 48 hours after the onset of pyrexia (>41°C) or immediately when other signs (apathy, anorexia, diarrhea) were present. Rabbits that did not develop pyrexia or clinical signs were euthanized at the termination of the experiment at 60 days PI. Necropsy was performed on all rabbits immediately after euthanasia and samples of lung, mesenteric lymph node, spleen, liver and appendix were snap frozen in liquid nitrogen, and later processed for DNA that was assayed for OvHV-2 DNA using hemi-nested and quantitative PCR [16] or fixed in 10% neutral buffered formalin for routine histological processing and examination.

#### OvHV-2 neutralizing activity of anti-CpHV-2 antibodies

Rabbits (n = 15) were inoculated with OvHV-2 that had been incubated with MCFV Ab- plasma from goats (n = 6) or MCFV Ab+ plasma from CpHV-2 infected goats (n = 6); all goats were confirmed to be uninfected with OvHV-2 using hemi-nested PCR. As a positive neutralization control three rabbits were inoculated with virus incubated with MCFV Ab+ plasma from an OvHV-2 infected sheep. Since both the sheep and goat MCFV Ab+ plasma cELISA endpoint titers were 320 both plasmas were used at a final dilution of 1:2 in the experiment. Virus preparation, sera/plasma treatment, inoculation, and sampling were done as described above.

### Statistical analysis

Differences between the mean AlHV-1 neutralizing titers of the pooled Ab samples was tested by one-way ANOVA, followed by Dunnett’s multiple comparisons test. Differences between survival curves in the OvHV-2 neutralization assay were determined using a log-rank test. For all analyses P values ≤0.05 were considered statistically significant.

## Results and Discussion

This study used an AlHV-1 neutralization assay and the rabbit model of OvHV-2 infection to test for cross-reactivity of neutralizing antibodies from select MCFV-infected reservoir hosts against AlHV-1 and OvHV-2.

### Neutralization of AlHV-1

**Fig 1:**
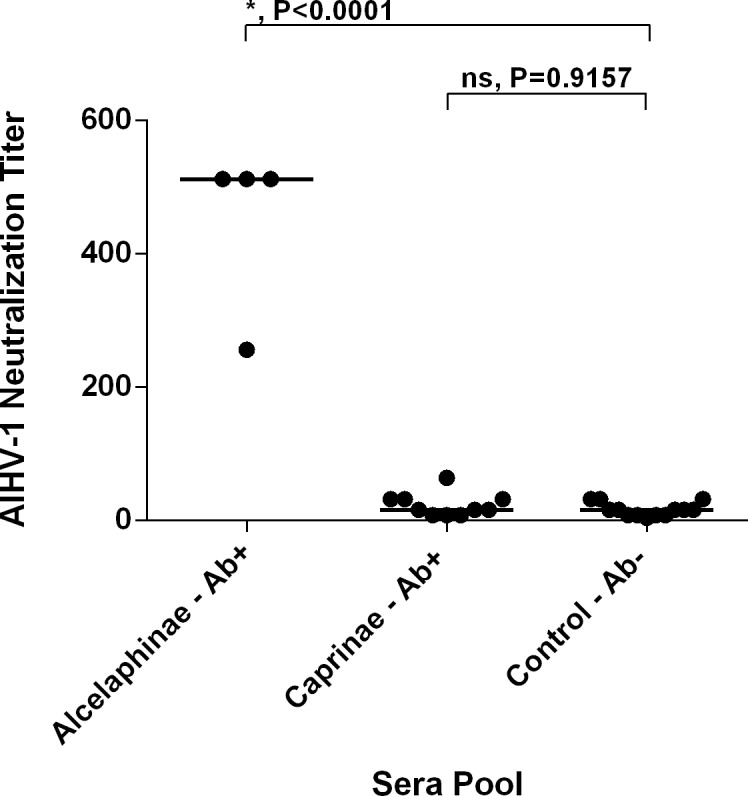
AlHV-1 neutralization by malignant catarrhal fever virus (MCFV) antibody-containing sera from animals in the subfamily Alcelaphinae. Virus neutralization titers are the reciprocal of the highest serum or plasma dilution which inhibited CPE in more than 50% of the wells at that dilution. Each circle represents a pool of sera. Lines indicate the mean titers in each group. * = Statistically significant difference. ns = not significant. Ab+ = MCFV Ab positive. Ab- = MCFV Ab negative.

In this report, pooled serum and plasma samples containing MCFV Abs, as measured by cELISA, from select species in the Alcelaphinae/Hippotranginae and Caprinae subfamilies were evaluated for AlHV-1 neutralizing activity (Table 1 and Fig 1). Matched control groups of pooled samples without detectable MCFV Abs as measured by cELISA were also assayed. The mean neutralizing titer of MCFV Ab + serum from animals in the subfamilies Alcelaphinae and Hippotraginae was 448±128, which was significantly greater than the mean titer of the MCFV Ab- control group (16.3±9.9; P<0.0001) (Fig 1). In contrast, pooled MCFV Ab+ samples from animals in the subfamily Caprinae had a mean neutralization titer of 23.2±17.5, which was not significantly different from the negative control group (P = 0.9157) (Fig 1). These data clearly show that samples from infected hosts in the Alcelaphinae and Hippotraginae subfamilies contain neutralizing antibodies against AlHV-1, but samples from hosts in the Caprinae subfamily do not.

Various serological assays, reviewed in Li, et al. [2], have been developed to test for anti-MCFV Abs. All of the assays use AlHV-1 as the antigen, therefore to detect seroconversion in animals infected with MCFVs other than AlHV-1, development of antibodies which cross-react with AlHV-1 needs to be present. Anti-AlHV-1 neutralizing Abs have been consistently detected in animals in the Alcelaphinae and Hippotraginae subfamilies [11, 17]. Sheep serum, however, has been reported to contain low to no AlHV-1 neutralizing activity [17, 18, 19] other than one study which reported neutralizing titers as high as 256 [20]. In that study 154 sheep sera were tested for neutralizing antibodies against the WC11 strain of AlHV-1; titers ≥ 4 (range 4–256) were reported as positive. Negative and positive control sera were used in the assay, but details such as the titers of the control sera and which species they were from were not provided, which makes it difficult to compare the studies. Based on our results the samples with low titers are likely displaying non-specific neutralizing activity.

### Neutralization of OvHV-2

To examine whether OvHV-2 neutralizing activity was present in MCFV Ab + serum from animals in the Alcelaphinae and Caprinae subfamilies, two *in vivo* OvHV-2 infection-protection experiments were conducted. These experiments are based on the fact that infection and development of MCF due to OvHV-2 are dependent on the dose of virus administered (reviewed in [2]). In rabbits, nasal secretion inocula containing 10^6^ OvHV-2 genome copies induce MCF while inocula containing 10^4^ genome copies fail to establish infection [12]. Incubation of inocula containing MCF-inducing doses of OvHV-2 with MCFV Ab+ plasma from OvHV-2 infected sheep fails to establish infection in rabbits due to an antibody-mediated reduction in the amount of virus below infectious levels [12, 21]

**Fig 2:**
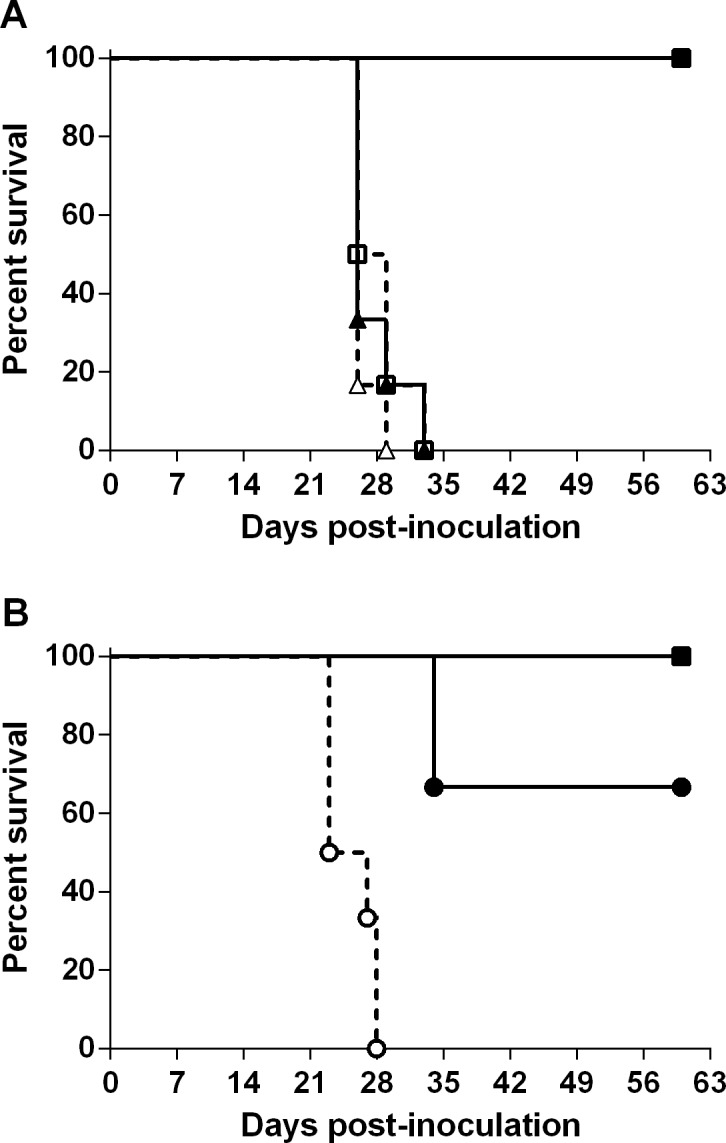
OvHV-2 neutralization by malignant catarrhal fever virus (MCFV) antibody-containing sera from goats, subfamily Caprinae. A) Survival curves of rabbits inoculated with OvHV-2 incubated with sera from wildebeest (triangles) or plasma from sheep (squares). B) Survival curves of rabbits inoculated with OvHV-2 incubated with sera from goats (circles) or plasma from sheep (squares). Closed symbols represent rabbits inoculated with OvHV-2 incubated with MCFV antibody positive sera (Ab+); open symbols represent rabbits inoculated with MCFV antibody negative sera or plasma (Ab-).

The first experiment examined whether MCFV Ab+ serum from AlHV-1 infected wildebeest prevented OvHV-2 infection of rabbits. All (6/6) the rabbits inoculated with OvHV-2 incubated with MCFV Ab + wildebeest serum became infected and developed MCF as did the control group inoculated with OvHV-2 treated with MCFV Ab- wildebeest serum (Table 1 and Fig 2A). As expected, all (6/6) control rabbits inoculated with OvHV-2 incubated with plasma from an OvHV-2 uninfected sheep became infected and developed MCF while none (0/6) of the rabbits receiving virus incubated with pooled plasma from OvHV-2 infected sheep became infected (Table 1 and Fig 2A). No significant differences were observed among the survival curves from rabbits inoculated with virus treated with wildebeest sera (Ab+ or Ab-) or sheep Ab–sera; however they were significantly different from the control group that received virus treated with Ab+ sera from sheep (P = 0.0002). Infection was confirmed by detection of OvHV-2 DNA in blood and tissues by PCR. Histopathological examination of tissues confirmed the presence of lesions associated with MCF (see S1 Fig for representative lesions). No viral DNA or lesions were detected in the tissues of rabbits that were healthy at the end of the experiment.

The second experiment was conducted to examine whether MCFV Ab+ serum and plasma from CpHV-2 infected goats prevented OvHV-2 infection of rabbits. Two out of six rabbits inoculated with OvHV-2 incubated with MCFV Ab + serum samples from CpHV-2 infected goats became infected and developed MCF (S1 Fig). No OvHV-2 DNA, MCFV antibodies, or MCF-associated histological lesions were present in the rabbits which did not develop disease. All (6/6) control rabbits inoculated with virus treated with MCFV Ab- samples from goats developed MCF (Table 1 and Fig 2B). None (0/3) of the control rabbits inoculated with OvHV-2 treated with MCFV Ab+ plasma from sheep became infected (Table 1 and Fig 2B). Statistical analyses indicated significant difference between the survival curves of rabbits receiving virus treated with Ab+ or Ab- goat sera (P = 0.0007); while no differences were detected when treatment with Ab+ goat or Ab+ sheep sera were compared (P = 0.2850).

The two rabbit experiments together showed that only samples from infected hosts in the Caprinae subfamily contained neutralizing antibodies against OvHV-2, which were able to block the virus and prevent infection and MCF in the rabbits. Samples from wildebeest at the single dilution used did not neutralize OvHV-2 to below disease-inducing levels; all rabbits inoculated with virus treated with wildebeest sera (Ab+ or Ab-) were infected and developed MCF. It should be noted that the *in vivo* assay requires at least a two log reduction in OvHV-2 from 10^6^ genome copies to 10^4^ copies to prevent infection, therefore it is possible that a low level of cross-reactivity of anti-AlHV-1 antibodies to OvHV-2 exists but is not detectable using this assay.

Both the *in vitro* and *in vivo* neutralization experiments demonstrated neutralizing cross-reactivity of Ab within an MCFV subgroup but not between subgroups. This information is critical to effectively use and interpret the diagnostic tests available for MCFVs. For instance, AlHV-1 neutralizing activity is an indicator of infection with MCFVs carried by reservoir animals in the Alcelaphinae and Hippotraginae subfamilies; however, the AlHV-1 neutralization test is not a suitable assay to detect infection with OvHV-2 or CpHV-2. Although not tested in this study, this may also be the case for other MCFVs carried by animals in the Caprinae subfamily.

Understanding how neutralizing antibodies cross react among MCFVs also has implications for vaccine development. Virion envelope glycoproteins gB, gH and gL are conserved among all herpesviruses and are attractive targets for vaccine-induced antibody neutralization. Indeed, rabbit hyperimmune sera generated against OvHV-2 envelope glycoproteins gB and the gL-gH heterodimer blocked OvHV-2 infection in the rabbit model [21]. Alignment of AlHV-1 and OvHV-2 gB, gH, and gL (S2 and S3 Figs) using the Constraint-based Multiple Alignment Tool (available at ftp://ftp.ncbi.nlm.nih.gov/pub/cobalt) showed gB to be more similar between the two viruses than gH-gL. Only incomplete sequences for CpHV-2 gB and no sequences for gH-gL are available, therefore alignments between AlHV-1 or OvHV-1 with CpHV-2 were not done. Given the alignment information, if the predominant neutralizing antibodies induced by MCFV infection in reservoir hosts were directed against gB we would expect sera from infected Caprinae to neutralize AlHV-1. Additionally, since OvHV-2 gB-specific antibodies block OvHV-2 infection in rabbits we would expect AlHV-1 infected wildebeest sera to also block OvHV-2 infection. However, this study yielded the opposite results. Therefore gB neutralizing antibodies in naturally infected hosts seem likely to be directed against non-conserved epitopes in AlHV-1 and OvHV-2. This means a vaccine that protects against MCF-induced by both viruses would likely need to contain neutralizing antigens from both viruses.

Neutralization of OvHV-2 infection by Ab+ serum from CpHV-2 infected goats shows that CpHV-2 and OvHV-2 share neutralizing antigens. This indicates CpHV-2 infection may be neutralized by anti-OvHV-2 antibodies. If this is the case, a vaccine which provides protection from OvHV-2 infection or MCF in clinically susceptible hosts might also be used to protect vulnerable exotic species in zoos and similar settings from CpHV-2 induced MCF.

## Supporting Information

S1 Fig Light microscopy of representative sections of liver. (DOCX)

S2 Fig Alignments of AlHV-1 and OvHV-2 glycoproteins L and H. (DOCX)

S3 Fig Alignment of AlHV-1 and OvHV-2 glycoprotein B. (DOCX)
