# Supplementary material for: Cross-Reactivity of Neutralizing Antibodies among Malignant Catarrhal Fever Viruses
Source: PLoS One. 2015 Dec 14;10(12):e0145073. doi: 10.1371/journal.pone.0145073 (PMC4681746; doi:10.1371/journal.pone.0145073)

S1 Figure. Light microscopy of representative sections of liver. Sections are stained with hematoxylin and eosin and black reference bars = 75 µm. A) Normal liver. B) Typical MCF lesions in liver. Severe portal inflammation (ateriolitis/phlebitis). Inset B) Portal venule with phlebitis (black arrow).


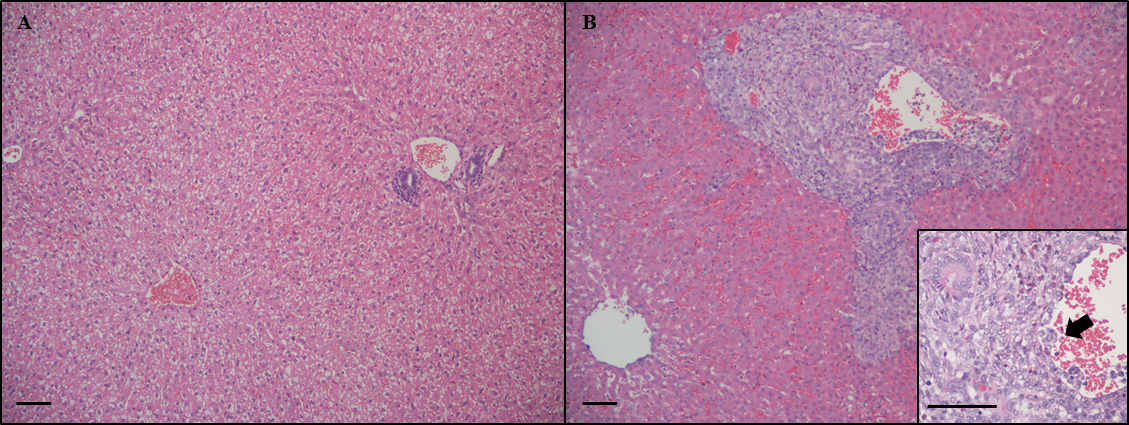

Supplement: S1 Fig — (DOCX) [file pone.0145073.s001.docx]
