# Supplementary material for: Cross-Reactivity of Neutralizing Antibodies among Malignant Catarrhal Fever Viruses
Source: PLoS One. 2015 Dec 14;10(12):e0145073. doi: 10.1371/journal.pone.0145073 (PMC4681746; doi:10.1371/journal.pone.0145073)
Supplement: S2 Fig — (DOCX) [file pone.0145073.s002.docx]

A

gi|10140968 1 MMWKWVTLLLFVLVCG---DNPVNAAAHNPFVCCHQKNETAHTPPKKAWSLVNAILHSPS

gi|83642882 1 -.ALLA.G.Y.L.G.SVLLSRVASVGSS...F....NST.L--..DILF.IT.I.F.A.K

gi|10140968 58 QCNHTNVALAYFNTTKGYKQVSCVNGFGLMSFCLALFDRLLTINVRVADQKFYDELLGYK

gi|83642882 58 ..QER...VV.V........AI.L...S....T...M.M..DTAYWLV.KR..N..AR..

gi|10140968 118 RGFAAQFSKATTDSSGFKNNLELDLITTRHGRMASKTHVAGLTRSSASSQL

gi|83642882 118 VQ.S.ELTAN.....M..TAFQSFTPRM.N.GV.-----------------

B

gi|10140944 1 --------MLFL---------ILLCVTGAQAITTPAPPRPATTTPRRGVTSAPLIVPASS

gi|83642859 1 MPSVPHAPL.WISHNPPAPGGL...FFTILSLWNN.------.GQ..PTI.EHY---.P.

gi|10140944 44 SELIVTLDGTFHSVTIDMTEIRQYVRQEIIEALWNASHVFESLETTYNRYKDVYRFTDQS

gi|83642859 52 .A.VI......Y.AQFRWED.EKII.RP..QD..KW.KTE.P.K...ET...I.K.PGK.

gi|10140944 104 IRVNTRGKLSTCKEVNKSTEVSFYKSITSQTINGKYDGDLGISNHQLGQQLFFYVMNVFP

gi|83642859 112 .K.PDGHQIGM.QPY.TTRQ.D.W.G..TA.VGDR.......FKN..R.E....IS....

gi|10140944 164 ----VENAFYPVRKHVVYSSLSLADGAYQLAGMATTNYVSLVVVRKISSTVTHEATIVFG

gi|83642859 172 PSHETHG......R.MI.T....DN.R.....V..I.......AYQV..V...S...I..

gi|10140944 220 NKKLLPSMRGSITKYDISLVNSDAEELLLLTSQKDYEYFSKNLFPQNWTDVFSLITSHTV

gi|83642859 232 DRLK.......VSRGE....YNN.........NT..A...Q....K..SE..TM..TN.A

gi|10140944 280 GELAQILQTSVVDFARKGRCRSVHFNSHFLTTYLAVLSLYYKMGTEFVSKNERQISLQCI

gi|83642859 292 DQ..VL....M..M...D...NI.I..Q..............I.M.MRYRSDFPV.....

gi|10140944 340 LPKLYEANVCFDMVHRCFTSQYTRGFDSDGINRLSAAILGSMPFEPNQGLSVPTNWFLQT

gi|83642859 352 ......TD..L......YVD......VTN.LH.......AAT..Y.E...E.TPD....N

gi|10140944 400 LYFVDGNLDPQNKGLHGITLILMDIYGRYVVNFTLTPEDRETLFYVYNALRGRKHLSTTM

gi|83642859 412

S2 Figure. Alignments of glycoproteins L and H. A) Alignment of AlHV-1 and OvHV-2 gL. B) Alignment of AlHV-1 and OvHV-2 gH. GenBank accession numbers gi|10140968 and gi|10140944 AlHV-1; gi|83642882 and gi|83642859 OvHV-2. Dots indicate identical residues. Dashes indicate gaps.
