# Supplementary material for: Cross-Reactivity of Neutralizing Antibodies among Malignant Catarrhal Fever Viruses
Source: PLoS One. 2015 Dec 14;10(12):e0145073. doi: 10.1371/journal.pone.0145073 (PMC4681746; doi:10.1371/journal.pone.0145073)
Supplement: S3 Fig — (DOCX) [file pone.0145073.s003.docx]

gi|10140934 1 MAHTGSTVCAFLIFAVLKNVFC--QTPTSSSEVEDVIPEANTVSDNIIRQQRN---NTAK

gi|83642848 1 ..SPA..LSSAALT.L.CLAA.LG....PPTTED...VPGH...PDVLK..LGSGL.EEE

gi|10140934 56 GIHS----DPSAFPFRVCSASNIGDIFRFQTSHSCPNTKDKEHNEGILLIFKENIVPYVF

gi|83642848 61 .SINRGPM.......................T............................

gi|10140934 112 KVRKYRKIVTTSTIYNGIYADAVTNQHVFSKSVPIYETRRMDTIYQCYNSLDVTVGGNLL

gi|83642848 121 .............V.....S..I....E......H..A.............SL.......

gi|10140934 172 VYTDNDGSNMTVDLQPVDGLSNSVRRYHSQPEIHAEPGWLLGGYRRRTTVNCEVTETDAR

gi|83642848 181 A......Y.L......M..........N...........................D.E..

gi|10140934 232 AVPPFRYFITNIGDTIEMSPFWSKAWNETEFSGEPDRTLTVAKDYRVVDYKFRGTQPQGH

gi|83642848 241 S.......V..V...........GGA....PNK..L..VS.LR..TL....D..SR..P.

gi|10140934 292 TRIFVDKEEYTLSWAQQFRNISYCRWAHWKSFDNAIKTEHGKSLHFVANDITASFYTPNT

gi|83642848 301 ....I...D........LK.............H.......EN.Y...........F....

gi|10140934 352 QTREVLGKHVCLNNTIESELKSRLAKVNDTHSPNGTAQYYLTNGGLLLVWQPLVQQKLLD

gi|83642848 361 EAQD.TKT.T...SL....MT...E...G..VT..SR......................N

gi|10140934 412 AKGLLDAVKKQQNTTTTTTTTRSRRQRRSVSSGI--DDVYTAESTILLTQIQFAYDTLRA

gi|83642848 421 .QD..E..ASKH.V.KPA---.......A...ILID........AL..........M..S

gi|10140934 470 QINNVLEELSRAWCREQHRASLMWNELSKINPTSVMSSIYGRPVSAKRIGDVISVSHCVV

gi|83642848 478 ...T........................................................

gi|10140934 530 VDQDSVSLHRSMRVPGRDKTHECYSRPPVTFKFINDSHLYKGQLGVNNEILLTTTAVEIC

gi|83642848 538 ...Q......N.......HA....................................L.V.

gi|10140934 590 HENTEHYFQGGNNMYFYKNYRHVKTMPVGDVATLDTFMVLNLTLVENIDFQVIELYSREE

gi|83642848 598 .........................I..SA.......I......................

gi|10140934 650 KRMSTAFDIETMFREYNYYTQRVTGLRRDLTDLATNRNQFVDAFGSLMDDLGVVGKTVLN

gi|83642848 658 .....V........................S.I............T............V.

gi|10140934 710 AVSSVATLFSSIVSGIINFIKNPFGGMLLFGLIAAVVITVILLNRKAKRFAQNPVQMIYP

gi|83642848 718 ....L........T.L............I...L.....A....R.R.AS..A........

gi|10140934 770 DIKTITSQREELQVDPISKHELDRIMLAMHDYHASKQ--PESKQDEEQGSTTSGPADWLN

gi|83642848 778 ..QQ..K..Q.MN.E..................QT..DK.DK.EGP.S.G-SANK.N...

gi|10140934 828 KAKNVLRRRAGYKPLKRTDSFESTGVP

gi|83642848 837 ............Q....S..T..AAAL

S3 Figure. Alignment of glycoprotein B. Alignment of AlHV-1 and OvHV-2 gB. GenBank accession number gi|10140934 AlHV-1; gi|83642848 OvHV-2. Dots indicate identical residues. Dashes indicate gaps.
